# Coping with Administrative Workload: a Pilot Study in the Usefulness of a Workshop for Psychiatric Trainees

**DOI:** 10.1007/s40596-023-01787-5

**Published:** 2023-05-01

**Authors:** Peter Deschamps, Asilay Seker, Marieke van der Schaaf, Marie-Aude Piot

**Affiliations:** 1 University Medical Centre Utrecht, Utrecht, The Netherlands; 2 Institute of Psychiatry, Psychology and Neuroscience, King’s College London, London, UK; 3 Utrecht Centre for Research and Development in Health Professions Education, University Medical Centre Utrecht, Utrecht, The Netherlands; 4 Université Paris Cité, Necker-Enfants Malades Hospital, Université Paris-Saclay, Paris, France

**Keywords:** Postgraduate training, Administration, Workload, Coping

## Abstract

**Objective:**

Administrative workload may have detrimental effects on medical postgraduate trainee satisfaction, capacity, and quality of care. Best-practice guidelines to help trainees cope have yet to be developed. This study explores perceptions of factors that influence the experience or amount of administrative workload at the personal and workplace level and evaluates the usefulness of a workshop on coping with this workload.

**Methods:**

A workshop was developed based on the Job Demands-Resources model, including a survey on perceptions of administrative workload; presentation on coping at personal (e.g., time management) and workplace (e.g., dealing with institutional rules) levels; personal plan of change during a group discussion; and reflective questionnaire after the session and again after 2 months. Perceptions of psychiatry trainee participants (*N* = 48) were collected.

**Results:**

Trainees estimated they spent half their time on administration (average 50%, *SD* = 15%). They wanted to spend less time (average 23%, *SD* = 11%) on most administrative duties, except for health record keeping. Personal factors that trainees experienced as helpful to cope included time management and analytical skills. Perfectionism was perceived as impeding. Supportive job factors included helpful supervisors, competent administrative staff, trust in a team, allocated timeslots, and information technology support. High workload and cumbersome procedures were mentioned as impeding. On average, trainees rated the workshop quality and the likelihood of bringing change to their practice with a 7 out of 10.

**Conclusion:**

Psychiatry trainees’ participation in a workshop on coping with administrative load during their training may be a worthwhile investment in the long term.

An increasing problem in postgraduate medical training practice is that trainees get discouraged and overwhelmed by spending too many hours on administrative duties. Clinicians in general at the beginning of their career hope to spend most of their time talking to patients, while reports suggest they spend 45 min on administrative tasks for every hour spent with patients [1]. A recent literature review showed consistency regarding the time spent by clinicians and their staff on billing and insurance-related activities (3 to 5 h per week) and quality measurement and reporting (potentially up to 15 h per week) [2]. Especially for early-career physicians, average time spent on administrative duties is associated with an increased likelihood of burnout [3]. A US nationwide survey of residents in postgraduate medical training found that the experienced workload created by clinical documentation may hamper optimal patient care as well as education [4].

Thus far, no best-practice guidelines provide guidance in this area. The present project represents a pilot study of the feasibility of a workshop to help psychiatry trainees cope with the administrative burden. The underlying theoretical framework for the workshop was based on the Job Demands-Resources model [5]. According to this model, every job includes qualitative demands (e.g., emotional or physical task-related aspects), quantitative demands (e.g., work overload or pace change), and organizational demands including bureaucracy. Resources can be job-related (e.g., support from others, job control, and performance feedback, which may enhance learning) or personal (e.g., knowledge and skills, resilience, optimism, goal-directedness) [5]. For the workshop, we focused on one specific demand: administrative workload, also referred to as administrative burden or bureaucracy. We aimed to help trainees to identify a potential discrepancy between their desired and actual administrative workload and to design a personal plan considering their own and environmental job resources.

Here, we report on the development and the content of the workshop. We evaluate the perceptions of the quantity and nature of administrative burden among psychiatry trainees who attended the workshop. We address the nature of perceived resources and demands at the personal and workplace level. We also report the trainees’ re-evaluation of the workshop 2 months later.

## Methods

The workshop was developed by three of the authors (PD, AS, MAP) based on the Job Demands-Resources model with the input of a panel of five psychiatry trainees provided by the European Federation of Psychiatric Trainees. The workshop included five steps, which Table 1 describes in more detail. Trainees filled in an online questionnaire at three time points. The questionnaire at the beginning of the workshop consisted of closed questions on the perceived and desired percentage of time spent on administrative duties and lists of options of tasks that were perceived as valuable and of tasks that were identified as a potential focus for improvement in coping. Four open questions invited reflection on personal and work-related factors that were perceived as helpful and harmful. The questionnaire at the end of the workshop investigated the usefulness of the workshop and the likelihood of change on a scale of 1–10. An open question asked trainees what they had appreciated most in the workshop. The questionnaire 2 months after the workshop included their estimated change in the percentage of time spent on administrative tasks, their perception of the likelihood of change at that time and in the future, and an open question to help them reflect on what they had learned.

**Table 1:** Workshop content, teaching method, and time schedule. Total workshop time = 1.5 h (listed timeframes are approximations). Parts 1–4 took place in one session; part 5 was performed 2 months later. *Points of data collection

Part 1: survey on perceptions of administrative workload (questionnaire; 15 min*)
• Actual and desired time spent on administrative tasks in % of total time
• Nature of administrative activities perceived as valuable in scope of training or patient care
• Nature of administrative activities in areas where change is desired
• Individual skills/resources and difficulties/pitfalls in obtaining a good balance
• Environmental resources and difficulties in obtaining a good balance
Part 2: potential coping options at various levels (performance; 30 min)
* Personal level*
• Work/life balance (e.g., notion that there is only one kind of time: your own time)
• Prioritization skills (e.g., awareness of what is most important)
• Time management (e.g., plan and do the large tasks first)
• Information technology skills (e.g., email management and dictation)
* Workplace level*
• Clarifying expectancies
• Delegating duties
• Making use of other’s strengths
• Having others help with your own weaknesses
* System level*
• from a behavioral learning perspective (e.g., explicit and consistent, ignore and reward)
• from a systems perspective (child versus parent mode, stronger together with other trainees)
Part 3: personal plan of change (in groups of 3–4 trainees; 30 min)
• Sharing of desired change: a shortlist of 3 administrative issues
• Discussing personal outcome measure for feedback later
• Discussion of resources (personal, technical and others) likely to help
Part 4: reflective questionnaire (at end of session; 15 min*)
• Action list to change own administrative burden after workshop
• Evaluation of workshop
Part 5: reflective questionnaire (at 2 months’ follow-up; 10 min*)

Responses to closed questions were descriptively analyzed (frequency, percentage, mean, standard deviation). Responses to open questions were analyzed using thematic analysis [6]; one of the authors sought systematic identification, interpretation, and developments of codes and themes derived from the content of the data [6].

Forty-eight participating psychiatric trainees volunteered with informed consent. They were sourced at three different occasions: at the local institution of the main author (*n* = 15 out of 24 available trainees), at a national congress of the Dutch Psychiatric Association (*n* = 20 during a parallel session), and at an international online training day organized by three international psychiatry training organizations (*n* = 13 during a parallel session). The follow-up was completed by 21 trainees after two email invitations.

## Results

At entry, trainees perceived the average time spent on administrative tasks compared to other clinical duties to be 50% (*SD* = 15%), while they thought that they should spend 23% on average (*SD* = 11%) on administration. Regarding the value of administrative tasks for training and education, trainees rated a medium average overall usefulness of these duties (on a scale of 1–7, *M* = 4; *SD* = 1.2). Trainees referred to several activities as valuable, mainly health record keeping, gathering own thoughts, and reflections on a case (implicitly, this referred to their written and not verbal activities within the context of the study) (*n* = 25) and “health record–keeping assuring continuity of care when you are not on call or for referral” (*n* = 18). Potential focuses for improvement included administrative duties related to health insurance processes (*n* = 16); arrangements with other departments/institutions (*n* = 11); legislation; forced care; and seclusion (*n* = 9); and emails or other sorts of communication (*n* = 10).

Personal skills and resources that aided the administrative workload included anticipating common tasks that would need their focus; prioritization of tasks; performing tasks efficiently; analytical skills, including the ability to summarize; and professional written communication skills (related both to patients and non-patients). Personal difficulties identified included dispersion (i.e., lack of selection between and within administrative tasks); perfectionism; overcontrol; and procrastination.

Helpful job-related factors included a supervisor who helped to stress the priority of direct patient contact and thinking how to organize administrative work accordingly, competent administrative staff that organized tasks smoothly, a trustable team that shared duties, management providing well-balanced assignments of clear tasks, technical support, and a well-structured agenda with daily timeslots allocated to administrative tasks. Job factors identified as impeding included too high an administrative workload; limited secretarial support; cumbersome procedures (e.g., legal procedures on involuntary admission and care); a lack of time for administrative tasks; and information technology/technical limitations.

After the workshop, trainees rated on a scale of 1–10 the usefulness of the workshop on average with a 7.5 (*SD*-1.1), the likeliness of change to their practice afterwards with an average of 7.1 (*SD* = 1.8) in the short term (i.e., the next weeks), and a 7 (*SD* = 1.5) in the long term (i.e., next months and years). Participants appreciated the support provided by the workshop to improve self-organization and prioritization. They realized they did not have to live up to every high-demanding standard and considered relying more on technical support and other team members. Overall, participants appreciated how the workshop fostered reflexivity and helped them experience more freedom of choice related to how they would manage tasks that were previously experienced as compulsory.

About half of the trainees (*n* = 21) responded to our invitation 2 months after the workshop. They estimated that the workshop on a scale of 1–10 was likely to have changed their practice over the past weeks, rating it with a 6.9 (*SD* = 1.8), and persistence of change over the following months/years was rated on average 7.1 (*SD* = 1.9). They reported to have spent on average 10% less time on administrative duties. Despite substantial variance in what they applied, all reported a struggle in their daily practice against wasting their time. All participants reported a new sense of control through concrete actions to get rid of administrative overload while improvement in technical and staff support was unequal.

## Discussion

The perception of psychiatry trainees who participated in the workshop was that they spend half their time on administration, in line with the literature that suggests that medical doctors spend up to 20 h/week on administrative duties [2]. Trainees identified several tasks that could be managed better. They thought that the administrative burden should be reduced by half. No comparison with previous studies that target individual trainees to cope with administrative burden is available. Another study that approached targeting interventions to change billing and insurance practices at the macro level suggested a roughly similar potential improvement to training and practice by reducing administrative burden by 30–50% [7].

The personal factors that trainees identified as helpful or as pitfalls in coping with administrative duties were mostly general and not specific to psychiatry training. They related to more general time- and self-management, issues that are likely to be shared with other (medical) professionals. Another part of the overall burden psychiatry trainees experience seems to relate to specifics, for example, psychiatric formulation writing and specific legislation that applies to the unvoluntary treatment of psychiatric patients [8].

The workplace-related factors that were perceived as relevant for coping with administrative tasks also included a general work overload and understaffing. The Job Demands-Resources model [5] suggests a clear distinction between quantitative demands (e.g., work overload or pace change) and qualitative demands (e.g., type of administrative tasks). Our results suggest that some trainees may be able to cope well with administrative tasks under low or medium workload conditions but may struggle with pace changes and workload increases. Thus, targeting both work overload and administrative burden at the same time may be needed. A study with interventions at the workplace level found positive effects of an experiment with better support by medical secretaries for internal medicine trainees and staff [9]. Of note, according to the perceptions of trainees and staff in that study, implementation was hindered by the unwillingness of individual doctors to delegate and to allow themselves to be temporarily unavailable, a theme that resonates with the perfectionism reported by trainees in the current workshop.

The evaluation of this pilot indicated a high appreciation of the elements of the workshop that focused on the personal and the workplace level but less appreciation of the parts that covered the overall system level. These system changes may well be out of the sphere of influence for most trainees. Some additional efforts may be needed to encourage trainees to reflect on system changes and prepare them for future cooperation with hospital staff and policymakers as they become seniors themselves.

A main limitation of the study was sample bias. The workshop was offered as self-selected or a voluntary parallel session during larger (inter)national events. No data were available to draw conclusions on the proportion of trainees that were attracted to the workshop. On one hand, it may well be that there is a portion of trainees who are not struggling or are not having difficulties with their administrative duties. On the other hand, there may be trainees who were too overwhelmed to participate and who felt they had to cope with the workload on their own. Such perceptions would resonate with the dimensions of perfectionism, overcontrol, and inability to delegate work found in our study. The broader literature suggests that high demands during medical school are to be cultivated as they prepare students to deal with the demanding nature of medical practice [10] and that students not strong enough to handle stress should probably give up medicine [11].

Another limitation is that our evaluation was based on the self-report of trainees and lacked an objective measure of actual time spent on administrative duties. More research is needed to further validate instrumentation to measure (perception of) administrative load. Preferably, objective data should be collected on the actual time spent on administrative tasks. To avoid these studies themselves increasing administrative burden, measures should preferably be deduced from already existing or automatic digital data. As the present study only succeeded in acquiring follow-up from a limited number of trainees, future studies should try to study the long-term effects of the intervention.

No data were collected on the stage in the training of participants. We were unable to explore differences in perception of administrative workload related to the overall experience level of trainees. Preferably, a workshop could be offered at the beginning of training. Those more experienced may also profit from help with coping with administrative duties. On one of the occasions the workshop was offered (at the national congress), a group of registered psychiatrists also took part in the workshop.

Finally, apart from international similarities across training, differences in, for example, health care, legislation, and insurance systems are also likely to play a role in the actual administrative workload, the perception of trainees, and the way they learn how to cope during their training, which may be an interesting focus for a larger international study.

In summary, the pilot showed the feasibility of a workshop to help trainees in psychiatry make a personal plan of change including personal and environmental factors with a modest investment of 2 h. The workshop was highly appreciated, and trainees expected it would bring change to their practice in the short and long term. Although the response to the follow-up questionnaire was too limited to allow for any firm conclusions, results from half of the attendees were encouraging. Further studies are required to evaluate whether the general themes that are relevant to reduce trainees’ administrative workload could be of value for trainees in other medical specialties as well. Trainers and training program directors whose curiosity has been triggered by the report of this pilot study are invited to request more details from the author group.

## Data Availability

Data available upon request.
